# Hyperthyroidism and vascular cell adhesion molecule-1 are associated with a low ankle-brachial index

**DOI:** 10.1038/s41598-020-74267-7

**Published:** 2020-10-13

**Authors:** Yu-Hsuan Li, I-Te Lee

**Affiliations:** 1 Division of Endocrinology and Metabolism, Department of Internal Medicine, Taichung Veterans General Hospital, #1650, Sec. 4, Taiwan Boulevard, Taichung, 40705 Taiwan; 2 Graduate Institute of Data Science, Taipei Medical University, Taipei, 11031 Taiwan; 3 School of Medicine, National Yang-Ming University, Taipei, 11221 Taiwan; 4 School of Medicine, Chung Shan Medical University, Taichung, 40201 Taiwan; 5 College of Science, Tunghai University, Taichung, 40704 Taiwan

**Keywords:** Cardiology, Endocrinology

## Abstract

We aimed to assess the ankle-brachial index (ABI) in patients with Graves’ disease. In the cross-sectional assessments, 81 patients with drug-naïve Graves’ disease and 235 with euthyroidism were enrolled. ABI and vascular cell adhesion molecule-1 (VCAM-1) levels were assessed. In the prospective follow-up, 32 patients with Graves’ disease were assessed again after antithyroid drugs for at least 4 weeks, and 32 age- and sex-matched controls with euthyroidism were also followed up. Patients with Graves’ disease had a higher VCAM-1 level (1309 ± 292 vs. 1009 ± 168 ng/mL, P < 0.001) and a lower ABI (0.98 ± 0.11 vs. 1.06 ± 0.10, P < 0.001) than those with euthyroidism. ABI was significantly lower in patients with hyperthyroidism and a high VCAM-1 level than in those with euthyroidism and a low VCAM-1 level (regression coefficient: − 0.050, 95% confidence interval [CI] between − 0.080 and − 0.019; P = 0.001). After treatment with antithyroid drugs, the change in free thyroxine (T4) level was inversely associated with the percentage change in ABI (regression coefficient: − 0.003, 95% CI between − 0.005 and − 0.001, P = 0.001). A synergistic effect of VCAM-1 and free T4 on ABI reduction was observed. After a longitudinal follow-up, an increase in ABI was significantly correlated with a decrease in the free T4 level.

## Introduction

Thyroid dysfunction is one of the leading causes of endocrine disorders^1–3^. Graves’ disease, characterized by autoimmune-associated hyperthyroidism, is the most common cause of excessive circulating thyroid hormone^4,5^. Thyroid hormones can directly act on cardiomyocytes and increase myocardial construction^6^. The risk of heart failure might increase as a result of changes in cardiac structure and hemodynamic regulation after long-term exposure to excessive thyroid hormone levels^7,8^.

Hyperthyroidism is associated with ischemic heart disease. Based on a Danish population-based cohort study, significantly increased risks of acute myocardial infarction and percutaneous coronary intervention were observed in patients after the diagnosis of hyperthyroidism^9^. Graves’ disease was also reported to increase all-cause mortality, cardiovascular (CV) mortality and CV events, including myocardial infarction, heart failure, ischemic stroke, and death^10,11^. Increased expression of vascular cell adhesion molecule-1 (VCAM-1) in endothelial cells has been reported in the thyroid gland of individuals with Graves’ disease^12^. Excessive thyroid hormone levels are associated with systemic inflammation and endothelial dysfunction^13,14^.

Peripheral artery disease (PAD) in the lower extremities is a manifestation of atherosclerosis and is associated with CV mortality^15,16^. The ankle-brachial index (ABI) is a noninvasive tool to clinically screen for PAD and a lower ABI value is associated with higher CV mortality^17–21^. Since thyroid hormones have extensive effects on peripheral arteries and the mechanism might be associated with the overexpression of VCAM-1 in endothelial cells^12,22–24^, we hypothesized that high circulating levels of thyroid hormone and VCAM-1 would be associated with low ABI values in patients with Graves’ disease.

## Materials and methods

### Patients and procedures

This study was conducted in the Division of Endocrinology and Metabolism in Taichung Veterans General Hospital. Adults who visited our outpatient department due to a suspicion of thyroid problems were screened between September 2012 and December 2017. The inclusion criteria for the case group were (1) a high serum free thyroxine (T4) level (> 17.6 pg/mL), (2) a suppressed thyroid-stimulating hormone (TSH) level (< 0.4 μIU/mL), (3) Graves’ disease based on clinical manifestation, and (4) a TSH receptor antibody (TRAb) level > 1 U/L; the inclusion criteria for the control group were (1) nodular goiter, (2) a normal serum free T4 level, and (3) a normal serum TSH level. The exclusion criteria for both the case group and control group were as follows: (1) a history of pharmacological treatment for thyroid diseases; (2) a history of diabetes mellitus; (3) a history of coronary artery disease; (4) a history of end-stage kidney disease; (5) a history of severe systemic disease, such as malignancies or psychiatric disorders; (6) current acute or chronic infectious diseases; (7) current thyrotoxic crisis; (8) current autoimmune diseases other than Graves’ disease; and (9) current pregnancy. Based on the assessment at baseline, we also excluded patients who had (1) a fasting plasma glucose level ≥ 126 mg/dL, (2) thyrotoxicosis other than Graves’ disease, (3) an incomplete four-limb assessment of the ABI due to a known history of lower-extremity surgery or hemodialysis treatment, and (4) an ABI value > 1.40. The study complied with the Declaration of Helsinki and was approved by the Institutional Review Board of Taichung Veterans General Hospital. Written consent was obtained from each patient before the study procedures were performed (Clinical Trial Registration Number: NCT02886949, ClinicalTrials.gov).

### Procedure

This study included two parts of analytic assessments. For the case–control study at baseline, subjects underwent ABI assessments after an interview for the collection of medical information and anthropometric measurements. Blood samples were obtained for laboratory assessments after a fasting period ≥ 6 h. Within a week after laboratory assessments, patients attended another visit and were categorized into Graves’ disease and euthyroidism groups.

For the prospective observational study, 32 nonsmokers who started to use thionamide antithyroid drugs prescribed by the same research physician were consecutively invited in the case group for a follow-up assessment. We also followed up 32 age- and sex-matched nonsmokers in the control group. All of these invited subjects underwent anthropometric measurements, ABI assessments and blood sample collection after at least a 4-week period of follow-up.

### Laboratory measurements

Plasma glucose levels were determined using the oxidase–peroxidase method (Wako Diagnostics, Tokyo, Japan). Serum creatinine and lipid levels were determined using commercial kits (Beckman Coulter, Fullerton, USA). Serum TSH (normal range 0.4–4 μU/mL) and free T4 (normal range 7.9–17.6 pg/mL) levels were determined using chemiluminescent immunoassays (Siemens Healthcare Diagnostics Inc., Llanberis, United Kingdom). TRAb levels were determined using the chemiluminescent method (normal level < 1 U/L; Roche Diagnostics, Mannheim, Germany). Serum C-reactive protein (CRP) was determined using an ELISA kit (R&D Systems, Minneapolis, MN, USA). The serum VCAM-1 concentration was determined by ELISA (R&D Systems, Minneapolis, USA). The estimated glomerular filtration rate (eGFR) was calculated according to the Modification of Diet in Renal Disease equation^25^ as follows: 186 × (serum creatinine [mg/dL])^−1.154^ × (age [year])^−0.203^ (× 0.742, if female).

ABI assessment was performed in the supine position using the validated device (VP-1000 Plus; Omron Healthcare Co. Ltd., Kyoto, Japan) after patients rested for at least 5 min. The lower systolic blood pressure of the two ankles was recorded as the ankle pressure. ABI values were calculated by dividing the recorded systolic pressure of the ankle by the higher systolic pressure of both arms^17^. The brachial-ankle pulse wave velocity (baPWV) was automatically determined using the ABI device. The reproducibility of the ABI was examined by repeated assessments in 20 subjects. A highly positive correlation of ABI values (correlation coefficient [*r*] = 0.937, P < 0.001) was observed between the results of the first and second measurements. The 95% confidence interval (CI) was 0.004 ± 0.050 for the bias of the ABI between repeated measurements based on Bland–Altman plots.

### Statistical analysis

We present the mean ± the standard deviation (SD) for continuous variables and numbers with percentages (%) for categorical data. The clinical variables were tested for statistically significant differences using Student’s *t* tests for continuous variables between two groups and using χ^2^ tests for categorical variables. The change in each continuous variable within a group at baseline and after follow-up was analyzed by paired *t* tests. The association between ABI values and free T4 levels was determined using Pearson’s correlation test. A two-tailed P value < 0.05 was considered statistically significant. Statistical analysis was performed using SPSS 22.0 (IBM, Armonk, NY, USA).

## Results

### Case–control assessment

**Figure 1:**
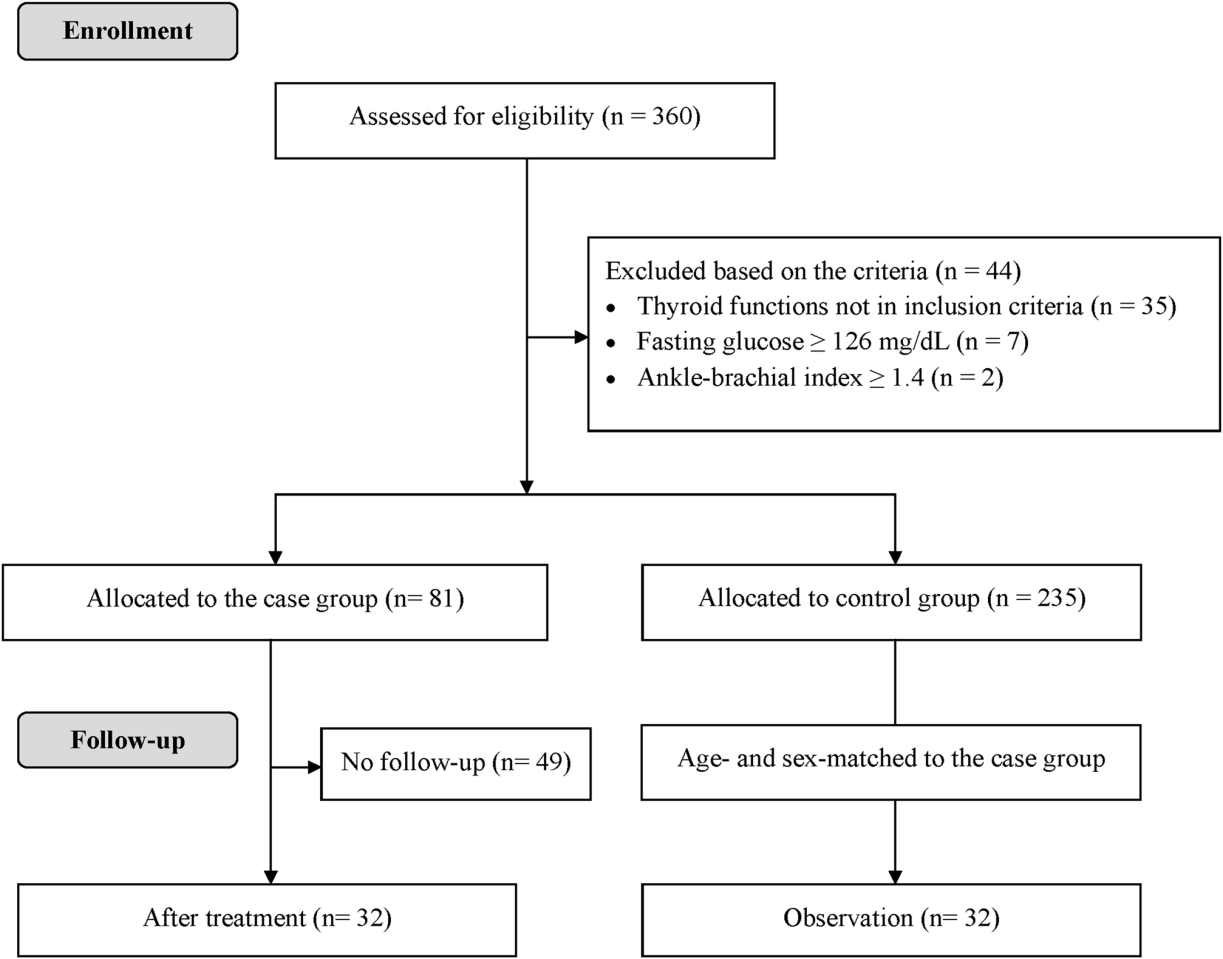
Flow diagram of the enrollment process for study subjects.

A total of 326 subjects were enrolled in this study. There were 81 subjects with Graves’ disease in the case group and 235 subjects with euthyroidism in the control group (Fig. 1). The median TRAb level was 30.6 U/L (interquartile range between 5.8 and 57.1 U/L) in the Graves’ disease group. The characteristics of these two groups are shown in Table 1. Patients with Graves’ disease had lower TSH and higher free T4 levels (both P values < 0.001) because of different inclusion criteria from those with euthyroidism. Furthermore, the subjects with Graves’ disease had a younger age (P < 0.001); a higher proportion of current smokers (P < 0.001); a lower body mass index (BMI) (P < 0.001); a higher brachial systolic blood pressure (P = 0.028); a higher heart rate (P < 0.001); a higher glucose level (P = 0.007); lower serum levels of total cholesterol (P < 0.001), high-density lipoprotein (HDL) cholesterol (P < 0.001), and triglycerides (P = 0.008); and a higher eGFR (P < 0.001) than those with euthyroidism. Notably, subjects with Graves’ disease had a lower systolic blood pressure at the ankle (125 ± 21 vs. 131 ± 22 mmHg, P = 0.018) and a lower ABI value (0.98 ± 0.11 vs. 1.06 ± 0.10, P < 0.001). Subjects with Graves’ disease also had a higher serum VCAM-1 level (1309 ± 292 vs. 1009 ± 168 ng/mL, P < 0.001).

**Table 1:** Clinical characteristics of the subjects with Graves’ disease and euthyroidism.

	Graves’ disease (n = 81)	Euthyroidism (n = 235)	P
Age (years)	37 ± 12	45 ± 14	< 0.001
Male, n (%)	15 (18.5%)	27 (11.5%)	0.156
Current smoker, n (%)	22 (27.2%)	12 (5.1%)	< 0.001
BMI (kg/m^2^)	21.7 ± 2.7	23.5 ± 3.9	< 0.001
Systolic BP (mmHg)	124 ± 15	120 ± 15	0.028
Diastolic BP (mmHg)	70 ± 9	71 ± 10	0.601
Heart rate (beats/min)	89 ± 16	74 ± 11	< 0.001
Fasting glucose (mmol/L)	5.2 ± 1.6	4.8 ± 1.1	0.007
Total cholesterol (mmol/L)	3.7 ± 0.8	4.8 ± 0.9	< 0.001
HDL cholesterol (mmol/L)	1.4 ± 0.3	1.5 ± 0.4	< 0.001
Triglycerides (mmol/L)	1.2 ± 0.7	1.6 ± 1.2	0.008
eGFR (mL/min/1.73 m^2^)	142.8 ± 45.6	95.0 ± 18.4	< 0.001
TSH (μIU/mL)	0.015 ± 0.043	1.547 ± 0.834	< 0.001
Free T4 (pg/mL)	37.1 ± 14.2	11.4 ± 1.6	< 0.001
ABI	0.98 ± 0.11	1.06 ± 0.10	< 0.001
**Categories of ABI, n (%)**			< 0.001
ABI ≤ 0.90	21 (25.9%)	12 (5.1%)	
ABI between 0.91 and 0.99	23 (28.4%)	58 (24.7%)	
ABI ≥ 1.00	37 (45.7%)	165 (70.2%)	
baPWV	1345 ± 204	1378 ± 239	0.270
Ankle systolic BP (mmHg)	125 ± 21	131 ± 22	0.018
VCAM-1 (ng/mL)	1309 ± 292	1009 ± 168	< 0.001
C-reactive protein (mg/L)	1.6 ± 4.5	1.5 ± 3.3	0.784

*ABI *ankle-brachial index, *baPWV *brachial-ankle pulse wave velocity, *BMI *body mass index, *BP *blood pressure, *eGFR *estimated glomerular filtration rate, *Free T4 *free thyroxine, *HDL *high-density lipoprotein, *VCAM-1 *vascular cell adhesion molecule-1, *TSH *thyroid-stimulating hormone.

**Figure 2:**
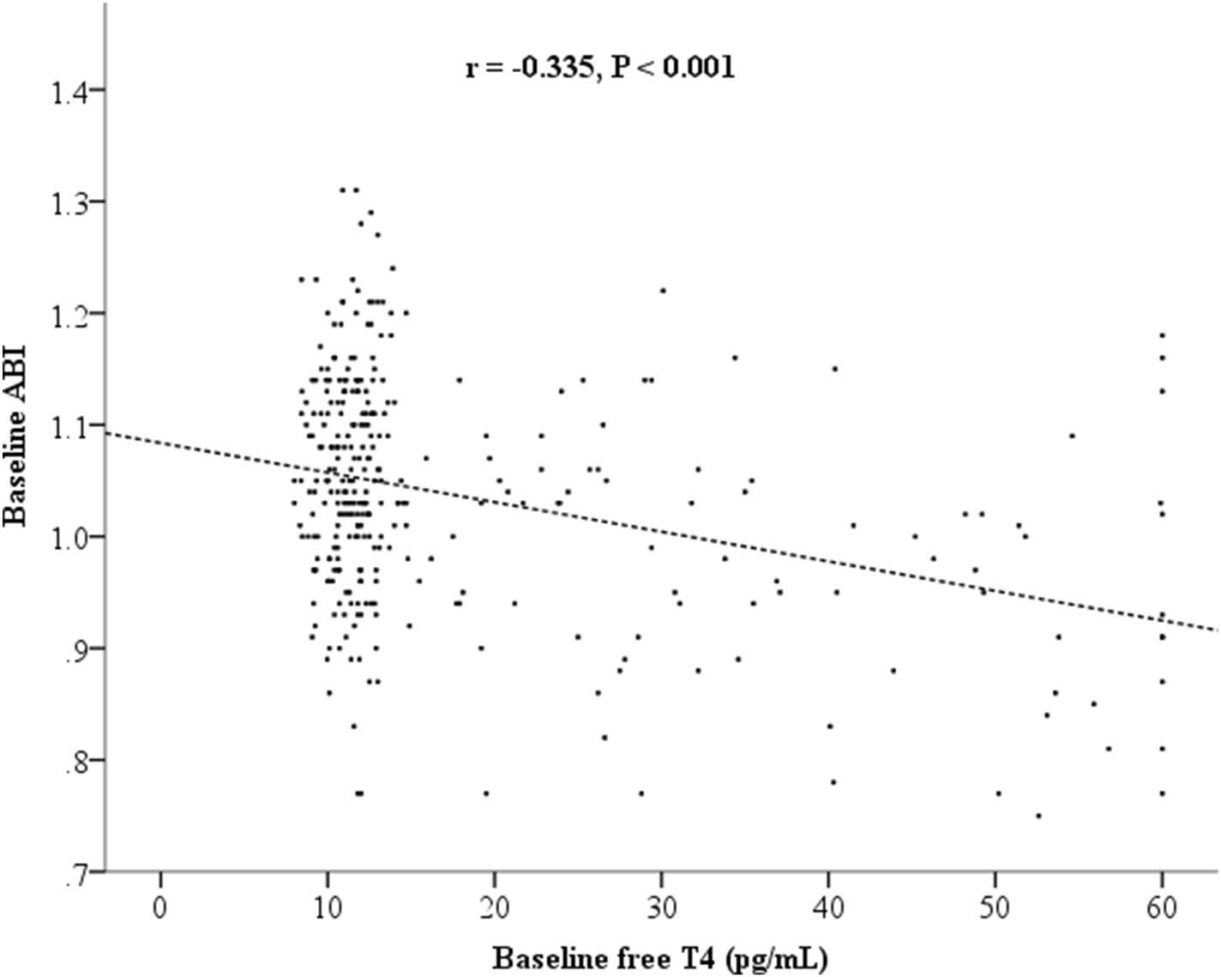
The correlation between the ABI values and free thyroxine (T4) levels at baseline (*r* = correlation coefficient).

We categorized the potential factors to assess the ABI value in this study (Table 2). The subjects with an older age, male sex, a higher BMI, a higher diastolic brachial blood pressure, a lower heart rate, a higher total cholesterol level, higher triglycerides, a lower eGFR, a higher baPWV, a lower serum VCAM-1 level, and higher CRP level had a higher ABI value than the other corresponding groups. Furthermore, ABI values were significantly correlated with free T4 levels (correlation coefficient [*r*] = − 0.335, P < 0.001; Fig. 2), but were not significantly correlated with CRP levels (P = 0.813).

**Table 2:** ABI values according to associated risk factors.

	n	ABI	P
Age*			< 0.001
< 41.4 years	158	0.99 ± 0.10	
≥ 41.4 years	158	1.08 ± 0.09	
**Sex**			0.002
Female	274	1.03 ± 0.10	
Male	42	1.08 ± 0.13	
**Current smoking**			0.101
No	282	1.04 ± 0.10	
Yes	34	1.01 ± 0.13	
BMI*			0.002
< 22.5 kg/m^2^	158	1.02 ± 0.10	
≥ 22.5 kg/m^2^	158	1.05 ± 0.10	
**Systolic BP**			0.701
< 120 mmHg	164	1.03 ± 0.10	
≥ 120 mmHg	152	1.04 ± 0.11	
**Diastolic BP**			0.001
< 70 mmHg	150	1.02 ± 0.11	
≥ 70 mmHg	166	1.05 ± 0.10	
Heart rate*			< 0.001
< 76 beats/min	156	1.07 ± 0.09	
≥ 76 beats/min	160	1.00 ± 0.11	
**Fasting glucose**			0.933
< 5.56 mmol/L	252	1.04 ± 0.11	
≥ 5.56 mmol/L	64	1.04 ± 0.11	
**Total cholesterol**			0.017
< 5.18 mmol/L	231	1.03 ± 0.11	
≥ 5.18 mmol/L	85	1.06 ± 0.09	
**Low HDL cholesterol**			0.051
No	246	1.04 ± 0.10	
Yes	70	1.01 ± 0.11	
**Triglycerides**			0.006
< 1.70 mmol/L	235	1.03 ± 0.11	
≥ 1.70 mmol/L	81	1.06 ± 0.10	
eGFR*			< 0.001
< 98.9 mL/min/1.73 m^2^	158	1.07 ± 0.10	
≥ 98.9 mL/min/1.73 m^2^	158	1.01 ± 0.11	
**baPWV**			< 0.001
< 1400 cm/s	198	1.02 ± 0.11	
≥ 1400 cm/s	118	1.07 ± 0.10	
VCAM-1*			0.010
< 1055 ng/mL	157	1.05 ± 0.10	
≥ 1055 ng/mL	159	1.02 ± 0.11	
C-reactive protein*			0.005
< 0.6 mg/L	159	1.02 ± 0.11	
≥ 0.6 mg/L	157	1.05 ± 0.10	

*ABI *ankle-brachial index, *baPWV *brachial-ankle pulse wave velocity, *BMI *body mass index, *BP *blood pressure, *eGFR *estimated glomerular filtration rate, *HDL *high-density lipoprotein, *VCAM-1 *vascular cell adhesion molecule-1, *TSH *thyroid-stimulating hormone.

*Cutoff value based on the median.

**Figure 3:**
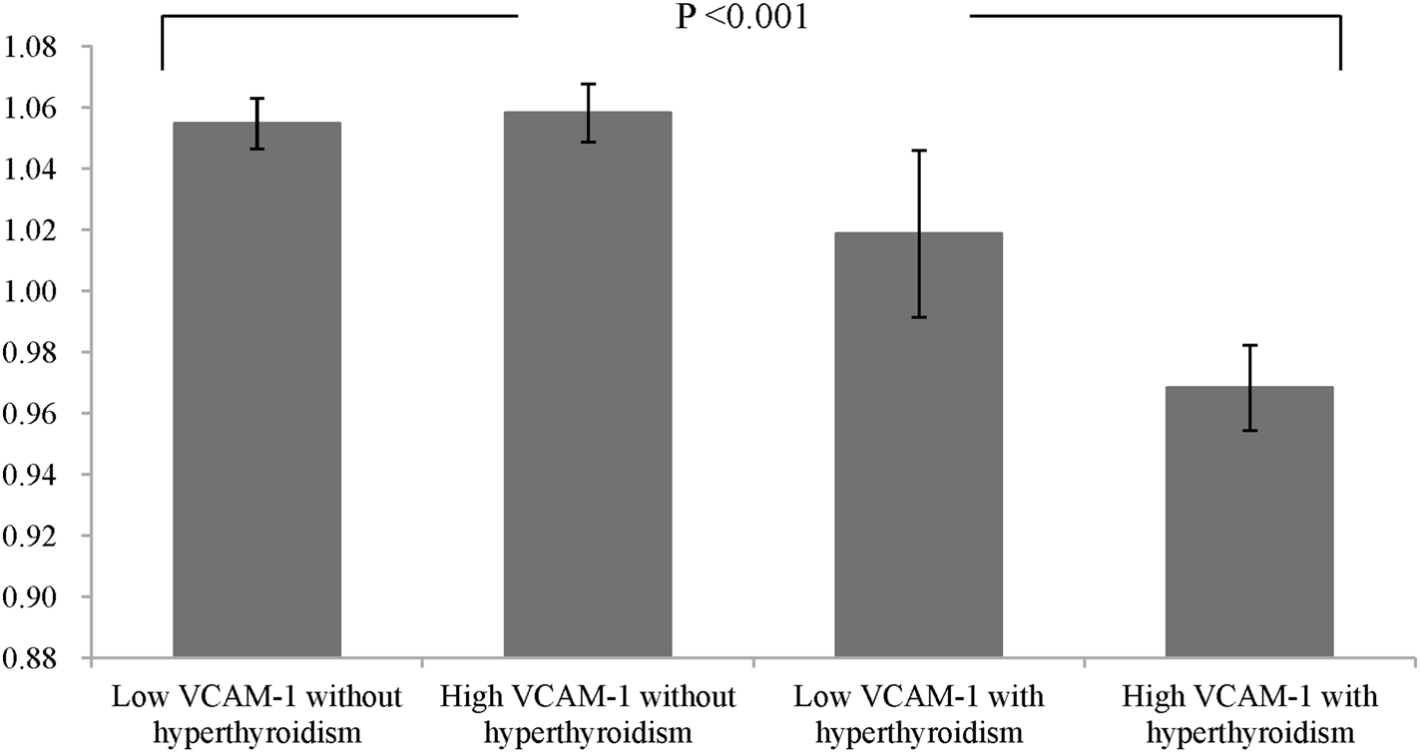
Ankle-brachial index (ABI) values are presented in the following groups: low vascular cell adhesion molecule-1 (VCAM-1) without hyperthyroidism, high VCAM-1 without hyperthyroidism, low VCAM-1 with hyperthyroidism, and high VCAM-1 with hyperthyroidism (P value for trend < 0.001). Patients were grouped according to hyperthyroid status and median serum VCAM-1 level (1055 ng/mL).

Because of the pathophysiological relationship between VCAM-1 and hyperthyroidism, we grouped all subjects into four groups: low VCAM-1 without hyperthyroidism (n = 142), high VCAM-1 without hyperthyroidism (n = 93), low VCAM-1 with hyperthyroidism (n = 15), and high VCAM-1 with hyperthyroidism (n = 66) according to the hyperthyroid status and the serum VCAM-1 median of 1055 ng/mL. As shown in Fig. 3, there was a significant reduction in the ABI value in the patients with hyperthyroidism (P value for trend < 0.001). In addition to age and sex, we selected the confounding factors as those variables which significantly influenced both hyperthyroidism status (shown in Table 1) and the ABI value (shown in Table 2) for the multivariate regression analysis. A high VCAM-1 level with hyperthyroidism was an independent risk factor for a low ABI value after adjustments for age, BMI, heart rate, total cholesterol, triglycerides, and eGFR (regression coefficient = − 0.050, 95% CI between − 0.080 and − 0.019; P = 0.001, Table 3).

**Table 3:** Multivariate regression analysis showing the factors associated with the ankle-brachial index (ABI) values.

	Crude			Model 1			Model 2		
	B	95% CI	P	B	95% CI	P	B	95% CI	P
**Low VCAM-1 without hyperthyroidism**	Ref.			Ref.			Ref.		
High VCAM-1 without hyperthyroidism	0.003	(− 0.023, 0.030)	0.793	− 0.010	(− 0.035, 0.014)	0.399	− 0.009	(− 0.033, 0.015)	0.450
Low VCAM-1 with hyperthyroidism	− 0.036	(− 0.089, 0.017)	0.184	− 0.031	(− 0.080, 0.018)	0.212	− 0.009	(− 0.058, 0.041)	0.728
High VCAM-1 with hyperthyroidism	− 0.086	(− 0.116, − 0.057)	< 0.001	− 0.078	(− 0.105, − 0.051)	< 0.001	− 0.050	(− 0.080, − 0.019)	0.001
Age ≥ 41.4 years				0.071	(0.050, 0.092)	< 0.001	0.065	(0.043, 0.087)	< 0.001
Male				0.052	(0.022, 0.082)	< 0.001	0.046	(0.016, 0.077)	0.003
BMI ≥ 22.5 kg/m^2^							0.008	(− 0.013, 0.029)	0.450
Hear rate ≥ 76 beats/min							− 0.047	(− 0.069, − 0.026)	< 0.001
Total cholesterol ≥ 5.18 mmol/L							− 0.006	(− 0.030, 0.018)	0.641
Triglycerides ≥ 1.70 mmol/L							0.012	(− 0.012, 0.036)	0.320
eGFR ≥ 98.9 mL/min/1.73 m^2^							− 0.014	(− 0.037, 0.009)	0.239

*B* regression coefficient, *Model 1* adjusted for age and sex, *Model 2* adjusted for age, sex, and the risk factors selected from Tables 1 and 2, *ABI *ankle-brachial index, *BMI *body mass index, *CI *confidence interval, *eGFR *estimated glomerular filtration rate, *VCAM-1 *vascular cell adhesion molecule-1.

### Longitudinal assessment of the subjects who had undergone antithyroid treatment

In the case group, all 32 recruited subjects completed assessments after treatment with antithyroid drugs for least 4 weeks. The median follow-up duration was 77 days (interquartile range between 57 and 123 days). The baseline characteristics of cases who participated in the follow-up were not significantly different from those of cases who did not participate in the follow-up. In the 32 subjects in the control group, the median follow-up duration was 87 days (interquartile range between 35 and 121 days). Despite being matched for age and sex, the subjects in the control group had a lower heart rate (P < 0.001), a higher total cholesterol level (P < 0.001), a higher HDL cholesterol level (P = 0.024), a lower eGFR (P < 0.001), a higher ABI value (P < 0.001), and a lower serum VCAM-1 level (P < 0.001) at baseline than the 32 subjects in the case group (Table 4).

**Table 4:** Baseline clinical characteristics of the subjects with and without follow-up in the Graves’ disease group and the control group.

	Case without follow up (n = 49)		Case with follow up (n = 32)	Controls with follow up (n = 32)	
	Mean ± SD	P*	Mean ± SD	Mean ± SD	P^#^
Age (years)	38 ± 11	0.360	36 ± 13	41 ± 11	0.074
Male, n (%)	12 (24.5%)	0.156	3 (9.4%)	3 (9.4%)	0.999
BMI (kg/m^2^)	21.6 ± 2.6	0.711	21.8 ± 2.8	23.0 ± 3.9	0.154
Systolic BP (mmHg)	125 ± 15	0.811	124 ± 16	117 ± 13	0.081
Diastolic BP (mmHg)	71 ± 10	0.194	69 ± 9	70 ± 10	0.500
Heart rate (beat/min)	90 ± 16	0.499	88 ± 16	73 ± 12	< 0.001
Fasting glucose (mmol/L)	5.3 ± 1.9	0.267	4.9 ± 1.0	4.6 ± 0.9	0.174
Total cholesterol (mmol/L)	3.8 ± 0.8	0.053	3.5 ± 0.8	4.7 ± 0.8	< 0.001
HDL cholesterol (mmol/L)	1.4 ± 0.3	0.305	1.3 ± 0.3	1.5 ± 0.3	0.024
Triglycerides (mmol/L)	1.3 ± 0.7	0.085	1.0 ± 0.7	1.3 ± 1.2	0.234
eGFR (mL/min/1.73 m^2^)	137 ± 44	0.185	151 ± 48	91 ± 14	< 0.001
TSH (μIU/mL)	0.016 ± 0.051	0.756	0.013 ± 0.030	1.383 ± 0.861	< 0.001
Free T4 (pg/mL)	37.6 ± 13.9	0.672	36.2 ± 14.9	11.7 ± 1.5	< 0.001
ABI	0.99 ± 0.12	0.156	0.96 ± 0.10	1.05 ± 0.10	< 0.001
baPWV (cm/s)	1343 ± 177	0.907	1348 ± 242	1319 ± 157	0.565
Ankle systolic BP (mmHg)	124 ± 19	0.340	120 ± 22	125 ± 18	0.352
VCAM-1 (ng/mL)	1305 ± 332	0.902	1314 ± 221	982 ± 168	< 0.001
C-reactive protein (mg/L)	1.2 ± 2.0	0.248	2.4 ± 6.8	1.0 ± 1.8	0.281

The control group included baseline euthryoid subjects matched for age and sex to cases with follow-up.

*ABI *ankle-brachial index, *baPWV *brachial-ankle pulse wave velocity, *BMI *body mass index, *BP *blood pressure, *eGFR *estimated glomerular filtration rate, *Free T4 *free thyroxine, *HDL * high-density lipoprotein, *VCAM-1 *vascular cell adhesion molecule-1, *TSH *thyroid-stimulating hormone.

*P: case without follow-up vs. case with follow-up.

^#^P: case with follow-up vs. control with follow-up.

After treatment with antithyroid drugs, free T4 significantly decreased (− 22.189 pg/mL, 95% CI between − 27.347 and − 17.031 pg/mL), but TSH did not significantly increase (2.600 μIU/mL, 95% CI between − 1.713 and 6.912 μIU/mL). Significant increases in BMI, total cholesterol level, and HDL cholesterol level were observed, and significant decreases in systolic and diastolic brachial blood pressure and heart rate were also observed during the follow-up period. A significant increase in ABI values and a decrease in VCAM-1 levels were also observed (Table 5). The increase in ABI values was significantly correlated with a decrease in free T4 levels (*r* = − 0.406, P = 0.021). In the control group, only the eGFR significantly increased and the VCAM-1 level significantly decreased during the observation follow-up period. The association between changes in free T4 levels and ABI values was not significant (*r* = 0.074, P = 0.689) in the control group.

**Table 5:** Differences from baseline values after follow-up.

	Cases (n = 32)		Controls (n = 32)		P*
	Mean	95% CI	Mean	95% CI	
BMI (kg/m^2^)	0.548	(0.134, 0.963)	0.130	(− 0.125, 0.385)	0.084
Systolic BP (mmHg)	− 7.594	(− 11.616, − 3.572)	1.438	(− 2.104, 4.979)	0.001
Diastolic BP (mmHg)	− 3.063	(− 5.678, − 0.447)	1.094	(− 1.846, 4.033)	0.035
Heart rate (beats/min)	− 10.906	(− 17.582, − 4.230)	− 1.438	(− 4.408, 1.533)	0.010
Fasting glucose (mmol/L)	− 0.347	(− 0.800, 0.106)	− 0.052	(− 0.536, 0.431)	0.367
Total cholesterol (mmol/L)	0.908	(0.651, 1.166)	− 0.049	(− 0.255, 0.156)	< 0.001
HDL cholesterol (mmol/L)	0.265	(0.169, 0.360)	0.019	(− 0.043, 0.082)	< 0.001
Triglycerides (mmol/L)	0.252	(− 0.117, 0.622)	− 0.154	(− 0.513, 0.205)	0.113
eGFR (mL/min/1.73 m^2^)	− 15.963	(− 33.059, 1.133)	8.229	(4.009, 12.448)	0.007
TSH (μIU/mL)	2.600	(− 1.713, 6.912)	0.195	(− 0.267, 0.656)	0.262
Free T4 (pg/mL)	− 22.189	(− 27.347, − 17.031)	0.319	(− 0.046, 0.684)	< 0.001
ABI	0.090	(0.044, 0.136)	0.009	(− 0.025, 0.042)	0.005
baPWV (cm/s)	− 48.84	(− 110.69, 13.00)	− 13.34	(− 57.46, 30.78)	0.344
VCAM-1 (ng/mL)	− 301.1	(− 384.0, − 218.3)	− 101.1	(− 147.1, − 55.0)	< 0.001
C-reactive protein (mg/L)	− 0.132	(− 0.368, 0.104)	0.023	(− 0.011, 0.058)	0.183

*Denotes P values for differences between case and control groups.

*ABI *ankle-brachial index, *baPWV *brachial-ankle pulse wave velocity, *BMI *body mass index, *BP *blood pressure, *eGFR *estimated glomerular filtration rate, *Free T4 *free thyroxine, *HDL *high-density lipoprotein, *TSH *thyroid-stimulating hormone, *VCAM-1 *vascular cell adhesion molecule-1.

Using multivariate linear regression, treatment with antithyroid drugs was an independent factor associated with an increased percentage change in the ABI value (regression coefficient = − 0.003, 95% CI between − 0.005 and − 0.001, P = 0.001) during the follow-up of the study after adjustment for age, sex, change in BMI, and change in heart rate (Table 6).

**Table 6:** The effect of changes in free thyroxine levels on the percentage change in the ankle-brachial index.

	B	95% CI	P
Crude	− 0.004	(− 0.006, − 0.002)	< 0.001
Model 1	− 0.004	(− 0.006, − 0.002)	< 0.001
Model 2	− 0.003	(− 0.005, − 0.001)	0.001

*B *regression coefficient, *CI *confidence interval, *Model 1* adjusted for age and sex, *Model 2* adjusted for age, sex, change in body mass index, and change in heart rate.

## Discussion

Our main findings are that a lower ABI was observed in patients with hyperthyroidism and high circulating VCAM-1 levels. After treatment with antithyroid drugs, ABI values were significantly increased, and the increase in the ABI values was correlated with a reduction in the circulating free T4 level. A lower ABI was associated with a higher mortality risk in subjects with an ABI value < 1.1^21,26^, and an ABI value < 1.0 should be considered to indicate a borderline abnormal ABI, as there was an association with increased CV risk even when the ABI value was > 0.9^27^. In the present study, Graves’ disease was related to a significant decrease in ankle systolic blood pressure, resulting in a low ABI, as a predictor of CV disease.

Patients with Graves’ disease had a higher mean VCAM-1 level than those with normal thyroid function. It has been reported that a high circulating VCAM-1 level was detected in patients with Graves’ disease, and the level was positively correlated with the free T4 levels^28^. The increased VCAM-1 level resulted from enhanced expression in the endothelium due to lymphocyte infiltration in the thyroid gland^12,29,30^. However, VCAM-1 released in the blood might induce chronic inflammation and endothelial dysfunction in peripheral arteries^13^. De Ciuceis et al.^31^ reported an increased level of VCAM-1 and a decreased number of endothelial progenitor cells in peripheral blood during hyperthyroid status in patients with Graves’ disease. Endothelial dysfunction was also associated with an increase in asymmetric dimethylarginine in patients with Graves’ disease^32,33^.

In the present study, only the VCAM-1 level, but not the CRP level, was significantly increased in patients with Graves’ disease. In line with our finding, Burggraaf et al.^34^ reported that the CRP level was not significantly associated with thyroid function. Furthermore, the VCAM-1 level is reported to be strongly associated with PAD^24^. In the present study, high circulating VCAM-1 levels had a synergistic effect with hyperthyroidism on low ABI values. Therefore, VCAM-1 might be a mediator in the relationship between hyperthyroidism and PAD.

Antithyroid drugs have been the most popular therapy used to treat hyperthyroid status^35,36^. In the present study, the ABI values increased after treatment, with reductions in both free T4 levels and VCAM-1 levels. In line with our data, antithyroid drugs have been reported to reduce circulating VCAM-1 levels^28^, and improve endothelial function^14^. Furthermore, an increased number of endothelial progenitor cells and decreased biomarkers of platelet activity were also reported after hyperthyroidism treatment^31,37^.

To the best of our knowledge, the present study is the first report of the relationship between the ABI and Graves’ disease. However, there are some limitations in the present study. First, we did not examine the mechanism or causal relationship between hyperthyroidism and PAD. Second, Graves’ disease was newly diagnosed in all patients with hyperthyroidism. However, we did not know the definitive time when hyperthyroidism occurred before diagnosis due to the diversity of symptoms. Third, our findings may not be applicable to patients with diabetes who were excluded due to a potential noncompressible artery at the ankle^38,39^. Fourth, we assessed only a reversible ABI reduction after treatment with antithyroid drugs but not long-term outcomes, such as lower-extremity amputation. Finally, we did not compare the effects of different Graves’ disease treatments on the ABI. It was reported that additional radioiodine therapy was associated with low long-term mortality^11^. Further large-scale studies with controlled treatment are needed to investigate the relationship between hyperthyroidism and PAD.

In conclusion, a higher free T4 level is associated with a lower ABI value in patients with untreated Graves’ disease. A high circulating VCAM-1 level had a superimposing effect on the decrease in the ABI value during hyperthyroid status. After treatment with antithyroid drugs, an increase in ABI values accompanied by reductions in free T4 and VCAM-1 levels was observed.
